# Healthcare resource utilization burden associated with cognitive impairments identified through natural language processing among patients with schizophrenia in the United States

**DOI:** 10.1038/s41537-025-00628-8

**Published:** 2025-05-27

**Authors:** Jerome Vaccaro, Mona Nili, Pin Xiang, James K. Nelson, Cory Pack, Randall Thompson, Joe Vasey, Joseph Parks

**Affiliations:** 1 RightPath HC, Millerton, NY USA; 2 Boehringer Ingelheim Pharmaceutical Inc, Ridgefield, CT USA; 3 Veradigm Health, Raleigh, NC USA; 4 National Council for Behavioral Wellbeing, Washington, DC USA

**Keywords:** Schizophrenia, Neuroscience

## Abstract

While cognitive impairments in schizophrenia are well documented in research, their identification and impact in clinical practice remain less well understood, despite their association with high patient burden and impact on long-term functional outcomes. In this study we aimed to identify documented cognitive impairments using natural language processing (NLP) and to characterize treatment patterns and healthcare resource utilization (HCRU) among patients with schizophrenia. This US retrospective cohort study used electronic health records (EHR) linked to administrative claims data from January 2016 through February 2023. Adult patients (≥18 years) with at least two schizophrenia diagnosis codes were included. Cognitive impairments were identified by NLP. Patient characteristics were assessed in the 12 months preceding the index date (first documented schizophrenia diagnosis). Treatment patterns and HCRU were measured over the 12 months after index date. A total of 79,326 patients were enrolled in the EHR cohort and 19,974 (25.2%) had documented cognitive impairments. Impairments in “Reasoning and Problem Solving” were identified most often (70.4%) followed by “Working Memory” (27.1%) and “Attention and Vigilance” (19.2%). In the EHR cohort, 11,293 patients (14.2%) had linked claims. Patients with documented cognitive impairments had more HCRU including outpatient visits, psychosocial interventions, and all-cause healthcare claims than patients without documented cognitive impairments (all *p* < 0.001). Patients with cognitive impairments had greater psychiatric pharmacy utilization than those without cognitive impairments. These observational data add to the limited published literature on cognitive impairments in patients with schizophrenia in the US. The low documented identification of cognitive impairments in this study underscores the importance of improving recognition and documentation of this important domain of schizophrenia. The association of cognitive impairments with high healthcare utilization further emphasizes the need for better treatment options for patients with schizophrenia.

## Introduction

Schizophrenia is a chronic and complex mental health condition with a poor prognosis. There are an estimated 1.7 to 3.3 million US adults living with the disease, and less than 14% of patients will achieve a sustained recovery, defined as both symptomatic remission and improvement in social functioning for 2 years or more^1–4^. Adults living with schizophrenia have over twice the risk of a premature death compared to adults living without schizophrenia^5^ and it is estimated that up to 13% die by suicide, a higher rate than in the general population^6,7^.

Schizophrenia is characterized by a mix of positive, negative, and cognitive symptoms^7–10^. Positive symptoms are those noted by their “presence,” and include reality distortion symptoms such as hallucinations and delusions as well as thought disorders like disorganized speech^8–10^. In comparison, negative symptoms refer to the absence of or a reduction in healthy emotions and behaviors^11–13^. Cognitive impairments associated with schizophrenia refer to deficits across cognitive domains expressed through verbal fluency, attention and concentration deficits, memory dysfunction, and executive functioning^7,14,15^. These deficits impact problem-solving abilities, adaptability, and social interactions^14^. Compared to positive symptoms, both negative symptoms and cognitive impairments in schizophrenia are not as well understood in real-world clinical practice although both relate to high patient burden and significantly impact long-term functional outcomes like the ability to obtain and hold a job^16,17^.

Cognitive impairments may persist when other symptoms are in remission and contribute to deficits in functioning and other disability outcomes^11,15^. For people with schizophrenia who report having cognitive impairments, the functional losses include lower educational attainment and higher poverty and unemployment rates^16^. They also report a lower quality of life including lower perceived health status and more reported limitations in activities of daily living^16^. Further, a lower level of cognitive functioning for those with schizophrenia is associated with higher comorbidity burden and a greater risk of natural-cause mortality^16,18^. In addition to the burden on patients, this higher clinical burden may explain the increase in healthcare resource utilization and direct and indirect costs among adults with schizophrenia experiencing cognitive symptoms^19^.

Although the estimated prevalence of cognitive impairments in people with schizophrenia is around 80%, it may be under-recognized^20^. Given the high prevalence and functional burden of cognitive impairments in the schizophrenia patient population, it is important to identify and document it; however, assessment scales for these impairments are rarely used in clinical practice^21^. Awareness and documentation of cognitive impairments may facilitate the clinician in adjusting their communication style and treatment recommendations. This may help prevent non-adherence due to cognitive impairments and improve outcomes among individuals with schizophrenia.

Real-world evidence studies on cognitive impairments in people with schizophrenia are limited^16,19,22^. In 2023, the ICD-10 Coordination and Maintenance Committee added instructional notes to identify cognitive deficits (impairments) for people with schizophrenia, allowing for improved documentation of these symptoms, although it is unclear how quickly they will be incorporated into clinical practice^23^. Given this framework, our objectives for this study were two-fold. First, we aimed to use electronic health record (EHR) data to identify documented cognitive impairment among patients with schizophrenia using natural language processing (NLP) and to describe demographic and clinical characteristics of these patients. Second, we intended to link EHR data to insurance claims in order to characterize treatment patterns and health care resource utilization among patients with schizophrenia with or without cognitive impairments.

## Methods

### Study design and population

This US-based retrospective cohort study using EHR and claims data included adult patients (≥18 years) with a diagnosis of schizophrenia (ICD-9: 295.XX; ICD-10-CM: F20.x). Participants must have experienced two or more schizophrenia outpatient encounters on or after January 1, 2016, and at least 12 months of EHR activity prior to the first schizophrenia diagnosis (EHR cohort). Patients with a diagnosis of dementia or non-schizophrenia-related cognitive impairments (dementia, frontotemporal lobe disease, prion disease, autism spectrum disorder, epilepsy, intellectual disability, multiple sclerosis, Parkinson’s disease, stroke, or traumatic brain injury) at any time during the study period were excluded. For the second objective, the subgroup analysis with EHR data linked to administrative claims only included patients who were continuously enrolled in medical and pharmacy benefits (i.e., with available claims data) for ≥12 months after the index date (linked claims cohort). The index date was defined as the date of the first schizophrenia diagnosis between January 1, 2016 and February 28, 2022.

### Data sources

Data were sourced from the Practice Fusion (PF) and Next Gen EHR databases, components of the Veradigm® Health Insights ambulatory (outpatient) medical facilities throughout the US. We obtained these data in accordance with the Health Insurance Portability and Accountability Act (HIPAA) and were granted an exemption from Institutional Review Board oversight. The practice sizes in the data were small (1–5 providers) to medium (<20 providers) and most prevalently located in the South, Southwest, and Northeast of the US. The PF dataset includes available, unstructured clinical notes for searching and NLP. Notes in the EHR may capture signs, symptoms, observations, and assessments that can identify cognitive impairments not typically available in structured EHR data.

To create the linked claims cohort for purposes of the second study objective, the PF EHR data were linked to closed (fully adjudicated, full enrollment) claims data. De-identified EHR and claims records were linked using Datavant’s deterministic matching solution (Datavant is a data logistics company for health-care (Datavant.com). This solution utilized encrypted, protected health information derived from tokens in both data sources to facilitate patient linking of de-identified data. Claims records included procedural, medical, and pharmaceutical claims from approximately 150 US health insurance vendors. The linked data set contains the all-cause healthcare information of patients across the US who have claims in the claims data set and who have interacted with a healthcare provider using the PF EHR system (linked claims cohort).

Sociodemographic variables for the total EHR cohort included age, sex, race, ethnicity, census region, and payer type. Clinical variables were Charlson Comorbidity Index (CCI) score and comorbid conditions^24^, body mass index (BMI), and psychiatric comorbidities. The CCI score is a weighted composite score that predicts 1-year mortality based on the presence of select comorbidities. A high score indicates a higher risk of mortality. Sociodemographic characteristics were assessed at the index date. CCI score and comorbid component conditions were assessed over the 12-month pre-index period. The BMI value used was the closest recorded value to the index date within 12 months before or after.

The treatment pattern and all-cause healthcare resource utilization were measured over the 12 months after index date for the linked claims cohort. The treatment pattern variables were pharmacologic therapy (i.e., 1st and 2nd generation antipsychotics, mood stabilizers, anti-depressants) and psychosocial interventions (i.e., cognitive behavioral therapy, psychotherapy, family therapy, psychosocial rehabilitation and/or group therapy). The all-cause healthcare resource utilization variables were outpatient visits, inpatient admissions, duration of inpatient admissions, emergency department visits, pharmacy claims, relapse episodes, and non-pharmacological therapy visits. A relapse episode was defined as either an emergency room (ER) visit claim with any diagnosis of a psychiatric condition or an inpatient hospitalization claim with a primary diagnosis of schizophrenia of schizoaffective disorder^25,26^.

### Natural language processing analysis

Natural language processing (“NLP”) was used to identify cognitive impairments in the clinical notes for the entire EHR cohort. All unstructured texts from patient records during the study period were processed for review.

Prior to initiating the retrospective cohort study, a feasibility study using NLP was conducted to determine if there were a sufficient number of eligible patients to warrant a full study and to test an initial list of key terms for identifying cognitive impairments (Supplementary Table 1).

The steps of NLP to identify and extract cognitive impairments indicators were:

- Data preprocessing: in this initial step, Python scripts were used to remove hypertext markup language (HTML) tags from clinical notes, converting them into plain text. This process required writing code to identify and eliminate HTML-specific syntax, ensuring the retention of only the actual text content for subsequent analysis.
- Text segmentation: once the text was clean of HTML tags, natural language processing libraries in Python were used to segment the text into individual sentences. This sentence segmentation analyzed punctuation and syntax to accurately delineate the end of one sentence and the beginning of another.
- Keyword extraction: this process began with the creation of an initial cognitive impairments list by the research team, in collaboration with medical informatics specialists and clinical experts, using the MATRICS™ Consensus Cognitive Battery (MCCB™) to identify key terms^15^. The MCCB is a set of 10 standardized tests covering seven domains that assess cognitive function in people with schizophrenia and related disorders. The seven domains of the MCCB are: (1) Speed of processing; (2) Attention/vigilance; (3) Working memory; (4) Verbal learning; (5) Visual learning; (6) Reasoning and problem-solving; and (7) Social cognition.

This initial list served as a starting point for scanning the data. Upon analyzing the data with this preliminary list, additional phrases were identified that expanded upon the existing terms. These were then incorporated into the list. The additional phrases were subsequently converted into regular expressions patterns allowing for efficient and precise identification of specific keywords and phrases in the data. A set of regular expressions patterns corresponding to relevant keywords was established. Sentences meeting predefined criteria of negation, questions, goals, or references to family history were excluded from further analysis. These rules effectively filtered out sentences that did not qualify as present cognitive impairments, ensuring the study focused on relevant data.

Findings from the feasibility study were used to further refine the initial list of cognitive impairments. Terms related to the “Social cognition” domain were rarely documented in the clinical notes, leading to the decision to exclude it. The domains of “Visual learning and memory” and “Verbal learning” were combined as there was overlap between the keywords used for each, making it difficult to differentiate between the two domains. Based on these findings from the feasibility study, only five domains were used for the primary retrospective cohort study. A list of the key terms within each domain and their frequency in the feasibility study is provided in the supplement (Supplementary Table 2).

### Quality control

A manual review of a randomly selected subset of the processed data (*n* = 250 patients) was conducted to ensure the reliability of the analysis. In this step, the outcomes of the Python natural language processing analysis were compared with manual interpretations to verify the accuracy and effectiveness of the algorithms used. F1 scores were calculated for each category of cognitive impairments. An F1 score is an industry standard machine learning evaluation metric that measures a model’s performance^27^. It combines the precision and recall scores of a model and computes how many times a model made a correct prediction across the entire dataset. The F1 score is calculated as the harmonic mean of the precision and recall scores with the equation: F1 = 2 * (precision * recall) / (precision + recall). The resulting score ranges from 0–100%, with a higher F1 score denoting a more accurate classifier. An F1 score of >0.8 is considered good performance.

### Statistical analysis

Descriptive reporting was stratified by the presence or absence of cognitive impairments in the EHR cohort. Results were described as counts and percentages for categorical variables and measures of central tendency (mean, median, standard deviation [SD]). Simple statistical comparisons between cohorts were conducted using Chi-square and *t*-test. No *p* value adjustments were made for multiple comparisons.

This study used a negative binomial regression model for calculating the adjusted estimates of inpatient admission. All the sociodemographic (age, gender, race, and region) and clinical characteristics (CCI score, anxiety, bipolar disorder, depression, panic disorder, post-traumatic stress disorder, and substance use disorder) were included in the regression models for calculating adjusted estimates.

## Results

**Fig. 1:**
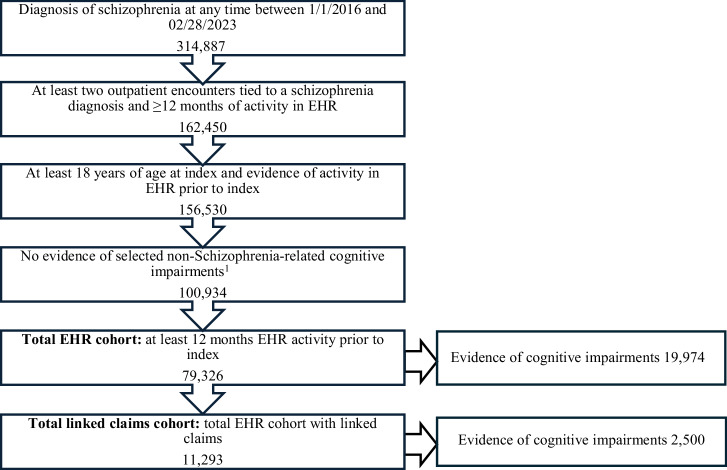
Study population. ^1^Evidence of stroke, dementia, prion disease, multiple sclerosis, or traumatic brain injury prior to index; or autism spectrum disorder, epilepsy, or intellectual disability at any time. EHR electronic health records.

Between January 1, 2016–February 28, 2023, 314,887 patients with schizophrenia were assessed for eligibility, of whom 79,326 (25.2%) met the selection criteria and were enrolled in the EHR cohort. Among the patients in the EHR cohort, 19,974 (25.2%) had documented evidence of cognitive impairments identified by NLP. Of the total patients in the EHR cohort, 11,293 (14.2%) had linked claims. Out of this linked-claims cohort, 2500 (22.1%) had cognitive impairments. The final study cohorts are shown in Fig. 1. Additional details on the selection criteria of the study population are provided in Supplementary Table 3.

### Demographic and clinical characteristics

Patients with documented cognitive impairments and patients without documented cognitive impairments in the EHR cohort and in the linked claims cohort were generally balanced in terms of age, gender, race, and BMI, although a greater proportion of patients with cognitive impairments in both the EHR and linked claims cohorts were non-Hispanic (70.8% vs. 47.3% (EHR) and 70.3% and 51.9% (linked claims), *p* < 0.001) compared to the cohorts without documented cognitive impairments. In the EHR cohort, patients with evidence of cognitive impairments had higher rates of psychiatric conditions including anxiety (18.9% vs. 15.3%), bipolar disorder (11.4% vs. 10.7%), post-traumatic stress disorder (5.4% vs. 4.1%), and substance use disorder (16.8% vs. 15.5%) compared to those without cognitive impairments (all comparisons *p* < 0.01). Similarly, in the linked claims cohort, patients with cognitive impairments also tended to have higher rates of psychiatric conditions including anxiety (39.0% vs. 28.8%), bipolar disorder (23.9% vs. 21.3%), depression (36.3% vs. 31.0%), panic disorder (4.3% vs. 2.6%), post-traumatic stress disorder (10.8% vs. 8.2%), and substance use disorder (33.4% vs. 30.8%) compared to those without cognitive impairments (all comparisons *p* ≤ 0.01). Table 1 shows the sociodemographic and clinical characteristics for study patients in the EHR cohort and in the linked claims cohort.

**Table 1:** Patient sociodemographic and clinical characteristics.

	EHR cohort					Linked claims cohort				
Variable	With documented cognitive impairments		No documented cognitive impairments		*p* value	With documented cognitive impairments		No documented cognitive impairments		*p* value
	*N* = 19,974		*N* = 59,352			*N* = 2500		*N* = 8793		
Age, years (mean, SD)	50.7	15.2	50.7	14.8	0.827	46.8	14.8	47.7	14.2	0.006
Age group (*N*,%)					<0.001					<0.001
18–44	7197	36.0%	20,763	35.0%		1138	45.5%	3674	41.8%	
45–64	8851	44.3%	27,781	46.8%		1071	42.8%	4178	47.5%	
65+	3926	19.7%	10,808	18.2%		291	11.6%	941	10.7%	
Gender (*N*,%)					0.029					0.466
Male	12,061	60.4%	36,408	61.3%		1550	62.0%	5522	62.8%	
Female	7902	39.6%	22,923	38.6%		950	38.0%	3271	37.2%	
Race (*N*,%)					<0.001					
White	9039	45.3%	25,824	43.5%		1083	43.3%	3667	41.7%	<0.001
Black	4366	21.9%	14,864	25.0%		522	20.9%	2429	27.6%	
Asian	989	5.0%	3139	5.3%		127	5.1%	493	5.6%	
Other	2920	14.6%	9344	15.7%		469	18.8%	1495	17.0%	
Unknown/Not reported	2660	13.3%	6181	10.4%		299	12.0%	709	8.1%	
Ethnicity (*N*,%)					<0.001					<0.001
Hispanic	939	4.7%	2732	4.6%		168	6.7%	521	5.9%	
Non-Hispanic	14,144	70.8%	28,098	47.3%		1757	70.3%	4563	51.9%	
Unknown/Not reported	4891	24.5%	28,522	48.1%		575	23.0%	3709	42.2%	
Region (*N*,%)					<0.001					<0.001
Northeast	2141	10.7%	7494	12.6%		320	12.8%	1159	13.2%	
Midwest	4461	22.3%	11,356	19.1%		394	15.8%	1431	16.3%	
South	5825	29.2%	16,507	27.8%		696	27.8%	2120	24.1%	
West	6549	32.8%	20,984	35.4%		1019	40.8%	3922	44.6%	
Other/Unknown	998	5.0%	3011	5.1%		71	2.8%	161	1.8%	
BMI (mean, SD)	30.1	6.4	30.2	6.3	0.005	29.7	6.5	30.1	6.3	0.004
CCI (mean, SD)	0.42	1.0	0.48	1.07	<0.001	0.80	1.5	0.80	1.43	0.935

	EHR cohort					Linked claims cohort				
Variable	With documented cognitive impairments		No documented cognitive impairments		*p* value	With documented cognitive impairments		No documented cognitive impairments		*p* value
CCI Conditions^a^ (*N*, %)										
HIV-AIDS	–	<1%	–	<1%	0.081	39	1.6%	144	1.6%	0.786
Any malignancy	261	1.3%	1042	1.8%	<0.001	57	2.3%	249	2.8%	0.134
Cerebrovascular disease	–	<1%	–	<1%		38	1.5%	146	1.7%	0.625
Chronic pulmonary disease	2348	11.8%	7807	13.2%	<0.001	540	21.6%	1913	21.8%	0.868
Congestive heart failure	443	2.2%	1697	2.9%	<0.001	102	4.1%	409	4.7%	0.225
Diabetes, chronic complications	1022	5.1%	3379	5.7%	0.002	237	9.5%	783	8.9%	0.376
Diabetes without complications	2800	14.0%	10,029	16.9%	<0.001	560	22.4%	2046	23.3%	0.363
Liver disease, mild	739	3.7%	2341	3.9%	0.122	208	8.3%	697	7.9%	0.523
Myocardial infarction	–	<1%	–	<1%	0.002	43	1.7%	173	2.0%	0.425
Peripheral vascular disease	755	3.8%	2161	3.6%	0.367	190	7.6%	545	6.2%	0.012
Renal disease	643	3.2%	2525	4.3%	<0.001	147	5.9%	477	5.4%	0.379
Rheumatic disease	–	<1%	–	<1%	0.328	50	2.0%	128	1.5%	0.054
Hypertension	4657	23.3%	16,833	28.4%	<0.001	1022	40.9%	3675	41.8%	0.413
Psychiatric conditions (*N*, %)										
Anxiety	3875	18.9%	9090	15.3%	<0.001	974	39.0%	2528	28.8%	<0.001
Bipolar disorder	2272	11.4%	6350	10.7%	0.008	597	23.9%	1869	21.3%	0.005
Depression	3593	18.0%	10,405	17.5%	0.142	907	36.3%	2725	31.0%	<0.001
Panic disorder	327	1.6%	725	1.2%	<0.001	108	4.3%	233	2.6%	<0.001
Post-traumatic stress disorder	1079	5.4%	2410	4.1%	<0.001	269	10.8%	721	8.2%	<0.001
Substance use disorder	3362	16.8%	9228	15.5%	<0.001	836	33.4%	2706	30.8%	0.011
Payer (*N*, %)					<0.001					0.006
Commercial	376	1.9%	922	1.6%		143	5.7%	392	4.5%	
Dual Medicare-Medicaid	420	2.1%	1230	2.1%		213	8.5%	746	8.5%	
Medicaid	3087	15.5%	9718	16.4%		1779	71.2%	6201	70.5%	
Medicare	395	2.0%	1354	2.3%		167	6.7%	686	7.8%	
Self-Insured	35	0.2%	94	0.2%		15	0.6%	37	0.4%	
Other	55	0.3%	273	0.5%		12	0.5%	89	1.0%	
Unknown	15,606	78.1%	45,761	77.1%		171	6.8%	642	7.3%	

*BMI* body mass index, *CCI* Charlson Comorbidity Index, *EHR* electronic health record.

^a^Only CCI conditions prevalent in ≥1% were included in the table.

### Identification of cognitive impairments in the EHR cohort

The F1 score of the NLP model was between 77% and 94% for the five domains of cognitive impairments indicating good performance of the developed model. The cognitive impairments domain identified most frequently in the EHR cohort was “Reasoning and Problem Solving” (70.4%). Documented cognitive impairments within the domains of “Working Memory” (27.1%), “Attention and Vigilance” (19.2%), “Verbal Learning and Memory” (16.6%), and “Speed of Processing” (15.1%) were found less frequently (Table 2).

**Table 2:** Most commonly occurring phrases or terms related to cognitive impairments in Schizophrenia within each main domain found in the clinical notes of the EHR cohort^a^.

Phrases or terms related to cognitive impairments in Schizophrenia within each main domain^b^	Patients identified with each type of cognitive impairment out of patients with evidence of any cognitive impairments in the EHR cohort, *N* = 19,974 (25.2%)
	*N* (%)
1. Attention and vigilance	**3840 (19.2%)**
Reduced attention	3242 (16.2%)
Poor concentration	829 (4.2%)
Difficulty understanding	130 (0.7%)
Difficulty staying focused	123 (0.6%)
2. Reasoning and problem solving	**14,057 (70.4%)**
Insight and judgment	13,935 (69.8%)
Learning new technology	96 (0.5%)
Difficulty learning	187 (0.9%)
Handling changes	73 (0.4%)
Manage bills money	132 (0.7%)
Planning	30 (0.2%)
Difficulty problem solving	6 (0.03%)
Difficulties with concrete thinking	31 (0.2%)
3. Speed of processing	**3009 (15.1%)**
Poverty of thought	1543 (7.7%)
Thought blocking	1078 (5.4%)
Participate in conversation	289 (1.4%)
Slow thinking	239 (1.2%)
Difficulty integrating thoughts, feelings, and behavior	244 (1.2%)
Ability to perform tasks	40 (0.2%)
Unable to do things quickly	10 (0.1%)
4. Verbal learning and memory	**3322 (16.6%)**
Difficulty expressing themselves	2151 (10.8%)
Hindered speech	1473 (7.4%)
Limited vocabulary	188 (0.9%)
Follow conversation	60 (0.3%)
Remembering what they are going to say	15 (0.1%)
5. Working memory	**5418 (27.1%)**
Poor memory	2821 (14.1%)
Remembering how to get to places	1435 (7.2%)
Difficulties remembering things	1263 (6.3)
Remembering names of people	516 (2.6%)
Remembering where they put things	25 (0.1%)
Remembering chores and responsibilities	8 (0.04%)

^a^NLP analysis was performed on the EHR cohort only.

^b^Cognitive impairments identified by NLP are not mutually exclusive. Patients may have experienced multiple terms listed in the clinical notes.

Overall, the most common terms or phrases identified were related to “insight and judgment” (69.8%) in the “Reasoning and Problem Solving” domain. Other terms found in more than 10% of patients with cognitive impairments were related to “reduced attention” (16.2%) in the “Attention and Vigilance” domain; “poor memory” (14.1%) in the “Working Memory” domain, and “difficulty expressing themselves” (10.8%) in the “Verbal Learning and Memory” domain. Within the “Speed of Processing” domain, the most common terms or phrases identified were related to “poverty of thought” (7.7%).

The number of clinical notes in the EHR was associated with the proportion of patients with documented cognitive impairments. In patients with >50 clinical notes, 52.3% had documented cognitive impairments; in patients with ≤5 clinical notes, 2.5% had documented cognitive impairments.

### Healthcare resource utilization

Table 3 presents the annual all-cause healthcare resource utilization for schizophrenia patients in the linked claims cohort with and without documented evidence of cognitive impairments. The length of hospitalization stay per admission was 15% higher for patients with cognitive impairments compared to those without (17.4 vs. 15.4 days, *p* value < 0.01). The mean per-patient per-year (PPPY) number of outpatient services was nearly 10% higher for those with cognitive impairments (45.0 vs. 40.3, *p* value < 0.01). Patients with cognitive impairments had more PPPY relapse episodes compared to those without documented cognitive impairments (0.43 vs. 0.39, *p* value = 0.03). More than 30% of patients with cognitive impairments were receiving psychosocial interventions compared to patients without documented cognitive impairments (36.6% vs. 27.4%, *p* value < 0.001); this difference was primarily due to the use of psychotherapy (32.9% vs. 24.0%, *p* value < 0.01). Of patients receiving psychosocial interventions, the mean PPPY number of days with a therapy session was 22% higher for those with cognitive impairments compared to patients without evidence of cognitive impairments (10.6 vs. 8.7, *p* value < 0.001).

**Table 3:** Annual healthcare resource utilization for patients in the linked claims cohort.

	Documented cognitive impairments	No documented cognitive impairments	*p* value
	*N* = 2500	*N* = 8793	
Annual healthcare resource utilization PPPY (Mean)			
Number of all-cause claims^a^	84.6	78.0	<0.001
Total hospitalized days	4.6	4.0	0.130
Length of stay per admission, days	17.4	15.4	<0.001
Number of hospitalizations	0.36	0.32	0.056
Number of emergency department visits	1.43	1.38	0.525
Number of outpatient visits	45.0	40.3	<0.001
Number of pharmacy claims	37.8	35.9	0.050
Number of relapse episodes	0.43	0.39	0.03
Receiving psychosocial intervention, %	36.6%	27.4%	<0.001
Cognitive behavioral therapy, %	0.2%	0.1%	0.174
Psychotherapy, %	32.9%	24.0%	<0.001
Family therapy, %	0.9%	0.7%	<0.001
Psychosocial rehabilitation, %	4.5%	4.0%	0.220
Group therapy/Counseling	3.7%	2.9%	0.049
Number of days with a psychosocial therapy session	10.6	8.7	<0.001
Any psychiatric pharmacy utilization, %	89.5%	82.1%	<0.001
First generation antipsychotics	19.8%	19.3%	0.542
Second generation antipsychotics	80.1%	70.9%	<0.001
Mood stabilizers	22.6%	18.4%	<0.001
Antidepressants	57.4%	50.8%	<0.001

*PPPY* per-patient per-year.

^a^Overall healthcare claims include emergency department, inpatient, outpatient, and pharmacy.

Patients with cognitive impairments had nearly 10% greater psychiatric pharmacy utilization than those without cognitive impairments (89.5% vs. 82.1%, *p* value < 0.001). This increased utilization was seen for second generation antipsychotics (80.1% vs. 70.9%), mood stabilizers (22.6% vs. 18.4%), and antidepressants (57.4% vs. 50.8%) (all *p* values < 0.001). Patients with cognitive impairments had 10% more annual all-cause healthcare claims (84.6 vs. 78.0, *p* value < 0.001).

The adjusted estimates for inpatient admission PPPY for patients with documented cognitive impairments were approximately 1.2 times higher than for those without documented cognitive impairments (0.38 vs. 0.32, *p* value < 0.001).

## Discussion

This is the first study to identify cognitive impairments in patients with schizophrenia using NLP in a large US EHR database, and it offers new insights into the characteristics of cognitive impairments in schizophrenia. In this study, 25.2% of patients in the EHR cohort and 22.1% of patients in the EHR and linked claims cohorts had documented cognitive impairments in their clinical notes. This is an overall lower proportion of patients with documented cognitive impairments compared to previous findings^16,22^. A recent analysis of data from the US 1997–2021 Medical Expenditure Panel Survey, which represents non-institutionalized individuals, revealed that cognitive impairments (57.6% cognitive limitations and 53.5% cognitive difficulties) were reported among over half of adults with schizophrenia^16^. Lipunova et al.^22^ found a similar prevalence were 59% of patients had a Mental Status Examination label indicating cognitive impairments in their clinical records in a retrospective EHR data study conducted in the US of 81,451 patients diagnosed with schizophrenia between 1999 and 2024^22^. Other reports indicate that neurocognitive dysfunction affects as many as 70–80% of individuals with schizophrenia^20,28^. The low identification of documented cognitive impairments in the clinical notes by NLP in this study does not necessarily mean that cognitive impairments were not present; instead, this discrepancy in prevalence may be in part due to the infrequent use of assessment scales in clinical practice^21^. Cognitive assessment scales typically used to identify cognitive impairments in schizophrenia have a lengthy administration time^29^. For example, the MCCB, a performance-based evaluation of seven key cognitive domains relevant to schizophrenia, takes over 1 h to administer^14^. Other cognitive assessments used in schizophrenia include the Brief Assessment of Cognition in Schizophrenia (BACS) and the Schizophrenia Cognition Rating Scale (SCoRS), both of which require approximately 30 min of administration time^30,31^. Due to time constraints, clinicians may prefer to complete only a diagnostic exam and give less focus to cognitive impairments when not using a structured assessment^29^. Rapid, accurate and valid assessment methods would better enable the identification of this important domain in patients with schizophrenia.

This study used the novel approach of an NLP algorithm based on the domains of the MCCB to identify cognitive impairments in people with schizophrenia. Documented evidence of cognitive impairments within the domain of “Reasoning and Problem Solving” (70.4% of patients with cognitive impairments) was found more than twice as frequently as the next most frequently found domain of “Working Memory” (27.1%), and over three times as often as the domain of “Attention and Vigilance” (19.2%). Specifically, within the domain of “Reasoning and Problem Solving,” keywords and phrases related to a patient’s “insight and judgment” (69.8%) were the most prevalent in clinical notes as detected through NLP. Other types of cognitive impairments related to “Reasoning and Problem Solving” such as difficulty learning, handling changes, managing bills and money, and planning were only 4% prevalent in the clinical notes. Likewise, within the second most frequent domain of “Working Memory,” the most commonly used terms found were related to “poor memory” although these terms were only present for around 14% of patients with documented cognitive impairments within this domain. Other terms used within the “Working Memory” domain but with less frequency included “remembering how to get to places” (7.2%), and “difficulties remembering things” (6.3%). The domain of “Attention and Vigilance” was most often described using terms related to “reduced attention” (16.2%). In comparison, deficits in “Social Cognition” such as difficulty talking to people and understanding feelings and facial expressions were rarely documented even though it is an important and consistently used domain in major cognitive test batteries and scales including the MCCB, and the Schizophrenia Cognition Rating Scale (SCoRS)^14,31^. Similarly, Mascio et al.^32^, using NLP in the United Kingdom, also found executive functioning deficits were the most frequently documented area of cognitive impairments (present in 53% of the patients), followed by memory (47%) and attention (44%)^32^.

A reported 80% of people with schizophrenia have cognitive impairments^20^. The findings of this study indicate that cognitive impairments, while common, are under-documented in patients with schizophrenia and inform the need for implementing accessible and efficient assessment tools in clinical practice, as cognitive impairments have important functional impacts. A cross-sectional analysis of the 1997–2021 Medical Expenditure Panel Survey found that adult schizophrenia patients reporting cognitive symptoms have higher work, house, school, social and recreational limitations and activities of daily living limitations as well as lower perceived mental and physical health status compared to those who did not report cognitive impairments^16^.

Our study adds to the limited real-world evidence studies conducted in the US focusing on cognitive impairments and the potential economic impact^16,17,19,22^. When we linked the EHR data to administrative insurance claims, we found a positive association between patients with schizophrenia who had documented, observed cognitive impairments and more relapse episodes and annual all-cause healthcare claims compared to study participants without cognitive impairments. This finding of increased healthcare utilization in patients with cognitive impairments was also reported by Choi et al.^19^ using MEPS data where cognitive impairments were associated with significantly increased healthcare resource utilization including both direct and indirect costs^19^. The number of annual per-patient outpatient visits ranged from 23.2 to 26.9 for those with cognitive limitations and cognitive difficulties, respectively, compared to 15.6 and 16.0 for those without cognitive impairments. Patients with cognitive limitations had nearly a 50% greater likelihood of an inpatient visit and 64% greater odds of emergency room visits.

In a study of 1135 patients with schizophrenia (mean age 40.0 years; 58.5% male), Kadakia et al.^33^ observed that patients with moderate to severe cognitive impairments had two times the number of relapse-related hospitalizations and ER visits leading to hospitalizations compared to those without cognitive impairments^33^. Similarly, Casso et al.^34^ reported a trend of patients with cognitive impairments having nearly twice as many ER visits compared to nonimpaired patients although the difference was not statistically significant (0.15 vs. 0.08)^34^. This greater resource utilization has a direct economic consequence as hospitalizations are the largest contributor to increased healthcare-related costs in this population^19^.

In this large, linked claims dataset, we did not see a difference in comorbidities between patients with and without cognitive impairments. This finding is different from recent studies reporting that schizophrenia patients reporting cognitive symptoms had higher comorbidities than those who did not report cognitive symptoms^19,35^.

Patients living with schizophrenia may experience positive, negative or cognitive symptoms, but FDA-approved pharmacological treatment options only target positive symptoms, with none effectively addressing cognitive impairments^36,37^. Over the past two decades, 52 new drugs were tested for cognitive impairment in patients with schizophrenia; of these, only two advanced to phase III^38^. This lack of approved treatments for cognitive impairment highlights the challenging need for continued research to improve outcomes for people living with schizophrenia. Guidelines from the American Psychiatric Association (APA) emphasize optimizing cognitive functioning and quality of life as important treatment-related goals^39^. Specifically, the APA suggests cognitive remediation therapy, which is designed to improve cognitive functions through repeated tasks. However, access to care is limited due to a shortage of mental healthcare professionals, particularly in rural areas of the US^40^. Adequate coverage is considered a population-to-psychiatrist ratio of 30,000 to 1 (20,000 to 1 where high needs are indicated)^41^. Using this definition, there are more than 6000 areas in the US designated as mental health professional shortage areas with some states disproportionately impacted more than others. Further, cognitive remediation therapy is not part of routine practice in the US, even where there is access to mental healthcare professionals. Vocational rehabilitation services are also not part of routine care in the US for schizophrenia^42^.

There are limited previous studies on the impact of cognitive impairments on patients with schizophrenia in the US. Our study demonstrates the successful use of NLP in EHR to identify cognitive impairments and offers new insights into the characteristics of cognitive impairments in schizophrenia and the resulting functional burden and clinical and economic impacts. The results of this study highlight the value of advanced data analytics of real-world data to provide a more comprehensive understanding of the patient experience with schizophrenia. A strength of this study is that it utilized a large sample size with over 79,000 schizophrenia patients in the EHR cohort and approximately 11,000 patients in the linked claims cohort.

Some important limitations should be considered. First, although this approach of using NLP facilitated an analysis that concentrated on text segments most relevant to identifying cognitive impairments in the clinical notes, it is possible and even likely that this process underestimated the identification of these symptoms experienced by patients with schizophrenia. One reason cognitive impairments may not be documented in the clinical notes is that because there are no approved treatments. In schizophrenia, available treatments focus on eliminating the positive symptoms of the disease and thus are more routinely mentioned, possibly leaving cognitive impairments, which are more difficult to assess, as less of a focus in clinical exam^29^. In the most basic scenario, if the clinician does not document the symptoms of cognitive impairments in the clinical notes, the NLP will not capture them. This study did show more documented cognitive impairments in patients with a greater number of clinical notes. This possibly indicates that providers who made more clinical notes were more inclined to document cognitive impairments. It is also possible that this association between cognitive impairments and greater healthcare utilization could be caused by patients with more healthcare visits having a greater likelihood of having clinical notes recorded but we could not determine causality in this observational study. Inherent in all observational research is the inability to infer causality. Instead, with this observational study design we intended to raise the question of how frequently cognitive impairments are documented for these patients and to describe characteristics of these patients.

The data sources for this study (the Veradigm Practice Fusion and Next Gen EHR databases) are used by primary care outpatient practices which may over-represent people with milder schizophrenia and less cognitive impairments compared to community mental health centers which may have a higher density of people with more severe schizophrenia and possibly greater cognitive impairment. This raises the question of whether the presumed missed information using the natural language processing approach is due to (1) the variability in the frequency and completeness of data collection, including the general lack of systemic approaches to collecting and documenting cognitive impairments in routine clinical practice; (2) the data source; (3) the natural language processing methodology itself; or (4) a combination of these factors.

Second, the study sample was not intended to be a random representative sample of US patients, but instead reflects the characteristics of health care providers who subscribe to the EHR platform. Therefore, patterns observed in these patients may not accurately portray all schizophrenia patients in the US. We acknowledge missing the undocumented people living with schizophrenia. The selection criteria may result in missing some prevalent cases that were diagnosed during the study period but the intent was to look at a large sample of clinical data to describe the presence of documented cognitive impairments and the characteristics of these patients. Further, patients included in this study were primarily located in the Southern and Western regions of the US. Additional large, geographically diverse studies may help to further understand the prevalence and characteristics of cognitive impairments on people living with schizophrenia in the US.

This study linking EHR data to claims data and using natural language processing is a novel approach to identifying cognitive impairments in patients with schizophrenia in the US and understanding the characteristics of patients impacted by these impairments. The low documented identification of cognitive impairments observed in this study underscores the importance of improving awareness, recognition, and documentation of this critical domain of schizophrenia. Despite the effort over the past two decades to understand and treat cognitive impairments in patients with schizophrenia, there has yet to be an approved therapy for this symptom domain of the disease. The increased healthcare resource utilization and burden associated with cognitive impairments in this study inform the need for innovative and effective treatment approaches to improve outcomes for patients living with schizophrenia.

## Supplementary information

Feasibility Study of Using Clinical Notes to Identify Cognitive Impairment in Patients with Schizophrenia

Additional Details on the Selection Criteria of the Final Study Population

## Data Availability

The data supporting the findings of this study are available from Veradigm® Health Insights. However, restrictions apply to the availability of these data for external sources, hence they are not publicly accessible. The data may be made available through the corresponding author upon a reasonable request.
