# Supplementary material for: Healthcare resource utilization burden associated with cognitive impairments identified through natural language processing among patients with schizophrenia in the United States
Source: Schizophrenia (Heidelb). 2025 May 27;11(1):82. doi: 10.1038/s41537-025-00628-8 (PMC12116770; doi:10.1038/s41537-025-00628-8)
Supplement: Supplementary file 1 — Feasibility Study of Using Clinical Notes to Identify Cognitive Impairment in Patients with Schizophrenia [file 41537_2025_628_MOESM1_ESM.docx]

**Supplement 1**

**Feasibility Study of Using Clinical Notes to Identify Cognitive Impairment in Patients with Schizophrenia**

**Objective**

The primary objective of this feasibility study was to determine if there were a sufficient number of eligible patients to warrant a full study of cognitive impairments among patients with schizophrenia. This was achieved using a multistep process. First, the study determined the number of eligible patients with and without clinical notes and with any neurological condition in order to establish the use of free-text documentation among patients regardless of note content. Second, the study aimed to identify eligible patients with notes that may be indicative of cognitive impairments associated with schizophrenia. This indicated the volume of notes containing potentially relevant data and the potential size of the full study sample. This feasibility study enabled us to conduct an in-depth demonstration of the NLP process for identifying and extracting cognitive impairment indicators from the clinical notes.

**Methods**

The key inclusion and exclusion criteria for the feasibility study matched that of the primary retrospective cohort study although with an observation period that began January 1, 2019 and without the requirement of 12 months or more of EHR activity prior to the first schizophrenia diagnosis.

**Results**

Between January 1, 2019 - February 28, 2023, 247,269 patients with schizophrenia were assessed for eligibility, of whom 41,816 (16.9%) met the selection criteria and had clinical notes. This feasibility study cohort is shown in Supplementary Table 1.

**Supplementary Table 1.** Feasibility Study Population

| **Selection criteria** | **N (%)** |
| --- | --- |
| Patients with a diagnosis of schizophrenia at any time within the study period | 247,269 |
| Patients with two or more outpatient encounters on or after start of study period, both of which must be tied to a schizophrenia diagnosis | 56,374 |
| Patients age ≥18 years at first schizophrenia encounter after start of observation period, January 1, 2019 | 55,828 |
| No evidence of non-Schizophrenia-related cognitive impairments^1^ | 41,882 |
| **Patients with clinical notes among total patients with a diagnosis of schizophrenia during study period** | **41,816** |
| **Evidence of cognitive impairments cohort** | **11,310** |
| No evidence of cognitive impairments cohort | 30,506 |

^1^Evidence of stroke, dementia, prion disease, multiple sclerosis, or traumatic brain injury prior to index; or autism spectrum disorder, epilepsy, or intellectual disability at any time

The cognitive impairment domain identified most frequently in the feasibility study cohort was "Reasoning and Problem Solving" (72.5%). Comparatively, documented cognitive impairments within the domains of "Working Memory" (30.4%), “Attention and Vigilance” (21.6%), “Verbal Learning and Memory”(19.5), and “Speed of Processing” (17.2%) were found less frequently (Supplementary Table 2).

Overall, the most common terms or phrases identified were related to “insight and judgement” (69.8%) in the “Reasoning and Problem Solving” domain. Other terms found in more than 10% of patients with cognitive impairments were related to “reduced attention” (16.2%) in the “Attention and Vigilance” domain; “poor memory” (14.1%) in the “Working Memory” domain, and “difficulty expressing themselves” (10.8%) in the “Verbal Learning and Memory” domain. Within the “Speed of Processing” domain, the most common terms identified were related to “poverty of thought” (7.7%).

**Supplementary Table 2** Most commonly occurring phrases or terms related to cognitive impairments in Schizophrenia within each main domain found in the clinical notes of the feasibility study EHR cohort

| Phrases or terms related to cognitive impairments in Schizophrenia within each main domain^1^ | Patients identified with each type of cognitive impairment out of patients with evidence of any cognitive impairment in the feasibility study cohort, N = 41,816 (16.9%) |
| --- | --- |
|  | N (%) |
| 1. **Attention and vigilance** | **1,886 (4.5%)** |
| Poor concentration | 741 (1.8%) |
| Reduced attention | 475 (1.1%) |
| Concentrating well enough to read newspaper / book | 279 (0.7%) |
| Difficulty understanding | 236 (0.6%) |
| Difficulty staying focused | 155 (0.4%) |
| 1. **Reasoning and problem solving** | **5,414 (12.9%)** |
| Poor insight and judgement | 4,119 (9.9%) |
| Poor insight (judgment not mentioned) | 325 (0.8%) |
| Poor judgement (insight not mentioned) | 105 (0.3%) |
| Manage specific tasks – bills, counting change | 364 (0.9%) |
| Difficulty learning new things | 172 (0.4%) |
| Handling changes | 164 (0.4%) |
| Learning how to use new gadgets and equipment | 76 (0.2%) |
| Planning ability | 47 (0.1%) |
| Difficulty problem solving | 22 (0.1%) |
| Difficulties with concrete thinking | 20 (0.1%) |
| 1. **Speed of processing** | **4,181 (10%)** |
| Thought blocking | 1,355 (3.2%) |
| Ability to perform tasks | 874 (2.1%) |
| Poverty of thought | 713 (1.7%) |
| Participate in conversation | 520 (1.2%) |
| Difficulty integrating thoughts, feelings, and behavior | 361 (0.9%) |
| Slow thinking | 351 (0.8%) |
| Unable to do things quickly | 17 (<0.1%) |
| 1. **Verbal learning and memory** | **4,038 (9.7%)** |
| Difficulty expressing themselves / thoughts | 2,417 (5.8%) |
| Hindered speech | 990 (2.4%) |
| Difficulty following conversation | 340 (0.8%) |
| Limited vocabulary | 233 (0.6%) |
| Difficulty remembering what they are going to say | 51 (0.1%) |
| Difficulty following TV | 7 (<0.1%) |
| 1. **Visual learning and memory** | **1729** (4.1%) |
| Poor memory | 1,727 (4.1%) |
| Visual attention | 2 (<0.1%) |
| 1. **Working memory** | **2,499 (6.0%)** |
| Poor memory | 1,727 (4.1%) |
| Difficulties remembering things | 361 (0.9%) |
| Remembering names of people | 168 (0.4%) |
| Remembering how to get to places | 105 (0.3%) |
| Remembering chores and responsibilities | 92 (0.2%) |
| Remembering where they put things | 46 (0.1%) |
| 1. **Social Cognition** | **1** (<0.1%) |
| Understanding facial expressions | 1 (<0.1%) |

^1^Cognitive impairments identified by NLP are not mutually exclusive. Patients may have experienced multiple terms listed in the clinical notes.
