# Supplementary material for: Healthcare resource utilization burden associated with cognitive impairments identified through natural language processing among patients with schizophrenia in the United States
Source: Schizophrenia (Heidelb). 2025 May 27;11(1):82. doi: 10.1038/s41537-025-00628-8 (PMC12116770; doi:10.1038/s41537-025-00628-8)
Supplement: Supplementary file 2 — Additional Details on the Selection Criteria of the Final Study Population [file 41537_2025_628_MOESM2_ESM.docx]

**Supplement 2**

**Additional Details on the Selection Criteria of the Final Study Population**

**Supplementary Table 3.** Study Population

| **Selection criteria** | **N (%)** | |
| --- | --- | --- |
| Patients with a diagnosis of schizophrenia at any time within the study period | 314,887 | |
| Patients with two or more outpatient encounters on or after start of study period, both of which must be tied to a schizophrenia diagnosis | 191,334 | |
| Patients with ≥12 months of activity in EHR during study period | 162,450 | |
| Patients age ≥18 years at index date | 161,019 | |
| Evidence of activity in the EHR at any time prior to index date | 156,530 | |
| No evidence of non-Schizophrenia-related cognitive impairments^1^ | 100,934 | |
| **Total EHR cohort: EHR activity ≥12 months prior to index** | **79,326** | |
| **Evidence of cognitive impairments cohort** | **19,974** |  |
| No evidence of cognitive impairments cohort | 59,352 | |
| **Total linked claims cohort: total EHR cohort with linked claims** | **11,293** | |
| **Evidence of cognitive impairments** | **2,500** | |
| No evidence of cognitive impairments | 8,793 | |

^1^Evidence of stroke, dementia, prion disease, multiple sclerosis, or traumatic brain injury prior to index; or autism spectrum disorder, epilepsy, or intellectual disability at any time

Abbreviations: EHR, electronic health records
